# Polyphosphate Reverses the Toxicity of the Quasi-Enzyme Bleomycin on Alveolar Endothelial Lung Cells In Vitro †

**DOI:** 10.3390/cancers13040750

**Published:** 2021-02-11

**Authors:** Werner E. G. Müller, Meik Neufurth, Shunfeng Wang, Heinz C. Schröder, Xiaohong Wang

**Affiliations:** ERC Advanced Investigator Grant Research Group at the Institute for Physiological Chemistry, University Medical Center of the Johannes Gutenberg University, Duesbergweg 6, 55128 Mainz, Germany; mneufurt@uni-mainz.de (M.N.); shunwang@uni-mainz.de (S.W.); hschroed@uni-mainz.de (H.C.S.)

**Keywords:** bleomycin, polyphosphate, pulmonary fibrosis, prevention of fibrosis, COVID-19, anti-SARS-CoV-2 activity

## Abstract

**Simple Summary:**

Bleomycin (BLM) is a medication introduced used to treat various types of cancer, including testicular cancer, ovarian cancer, and Hodgkin’s disease. Its most serious side effect is pulmonary fibrosis and impaired lung function. Using A549 human lung cells it is shown that, in parallel to an increased cell toxicity and DNA damage, BLM causes a marked enlargement of the cell nucleus. This effect is abolished by inorganic polyphosphate (polyP), if this physiological polymer is administered together with BLM. The detoxification of BLM is–most likely–caused by the upregulation of the gene encoding the BLM hydrolase which inactivates BLM in vitro and in vivo. This study contributes also to a rational application in COVID-19 patients since polyP prevents binding of SARS-CoV-2 to host cells.

**Abstract:**

The anti-cancer antitumor antibiotic bleomycin(s) (BLM) induces athyminic sites in DNA after its activation, a process that results in strand splitting. Here, using A549 human lung cells or BEAS-2B cells lunc cells, we show that the cell toxicity of BLM can be suppressed by addition of inorganic polyphosphate (polyP), a physiological polymer that accumulates and is released from platelets. BLM at a concentration of 20 µg ml^−1^ causes a decrease in cell viability (by ~70%), accompanied by an increased DNA damage and chromatin expansion (by amazingly 6-fold). Importantly, the BLM-caused effects on cell growth and DNA integrity are substantially suppressed by polyP. In parallel, the enlargement of the nuclei/chromatin in BLM-treated cells (diameter, 20–25 µm) is normalized to ~12 µm after co-incubation of the cells with BLM and polyP. A sequential application of the drugs (BLM for 3 days, followed by an exposure to polyP) does not cause this normalization. During co-incubation of BLM with polyP the gene for the *BLM hydrolase* is upregulated. It is concluded that by upregulating this enzyme polyP prevents the toxic side effects of BLM. These data might also contribute to an application of BLM in COVID-19 patients, since polyP inhibits binding of SARS-CoV-2 to cellular ACE2.

## 1. Introduction

The bleomycins (BLMs) are glycopeptides (size of ~1415 g/mol) with antineoplastic activity, which bind to DNA in a particular manner and cause strand-scissions in a reaction in which Fe(II) and oxygen are required [1]. It was the group of Umezama and colleagues [2] who first discovered these metallo-glycopeptide antibiotics in Streptomyces verticillis, purified them and determined their structures. Finally they revealed their mode of action in vitro [3], studied their biological efficiency in animal model [4] and brought them to the clinic as cancer chemotherapeutic agents [5]. In most clinical studies BLM is applied as a mixture of several bleomycins, predominantly consisting of BLM A2 and B2 [6].

**Figure 1:**
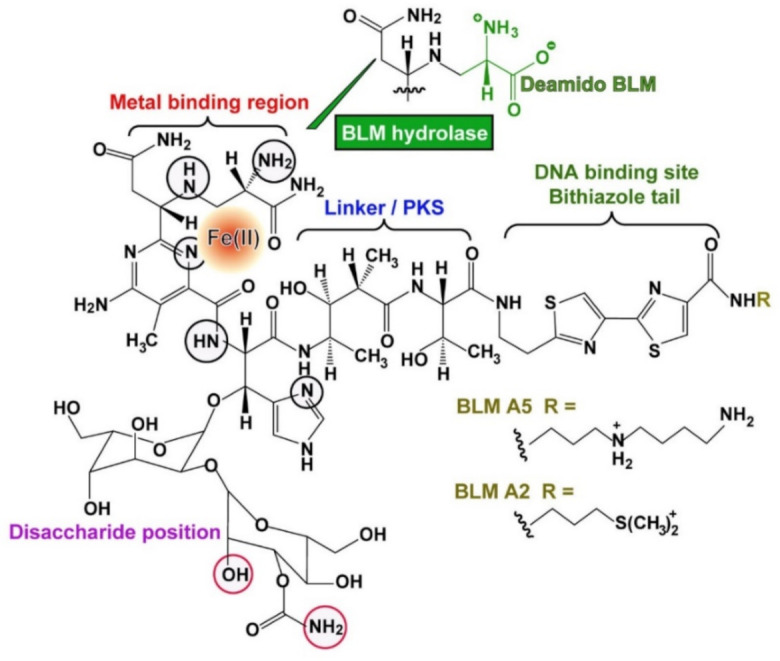
Metal (Fe(II))-BLM complex. Structure modified according to [8,9]. The modular built BLM molecule comprises the DNA binding site, the linker region synthesized by polyketide synthase, the metal binding region with its interacting nitrogen atoms (black circles) and the disaccharide portion (the red circles interact with the minor groove of the DNA). Fe(II) is the most active central transition metal ion. The BLM species A_5_ and A_2_ are present in the clinically used formulation. The reaction of the BLM hydrolase which catalyzes the reaction to the less toxic deamido BLM is outlined.

The drug is synthesized in the bacterium by modular non-ribosomal peptide synthetases and also polyketide synthases [7]. The compound can be dissected into several regions; the metal binding region, the linker with its small polyketide stretch, the DNA binding site and finally the disaccharide position [8] (Figure 1). The metal-binding domain involves a distorted square pyramidal arrangement, whereby the metal is fixed to the imidazole, deprotonated His amide, pyrimidine, and the primary and secondary amine of the β-aminoalanine residue [9]. The DNA recognition site has been narrowed down to the bithiazole moiety with its cationic tail, like shown for BLM A_5_ and A_2_ (Figure 1). A partial intercalation of BLM with DNA has been proposed even at the physiologically relevant temperature of 30 °C [8]. After binding the metal binding domain and disaccharide units partially stack against each other and display base-specific hydrogen bonding to the minor groove of the DNA [9]. Among a series of transition metal ions that bind to the nitrogen atoms of BLM, like Fe, Co, Cu, etc., the divalent Fe(II) cation is the most important one for causing biological activity [10].

**Figure 2:**
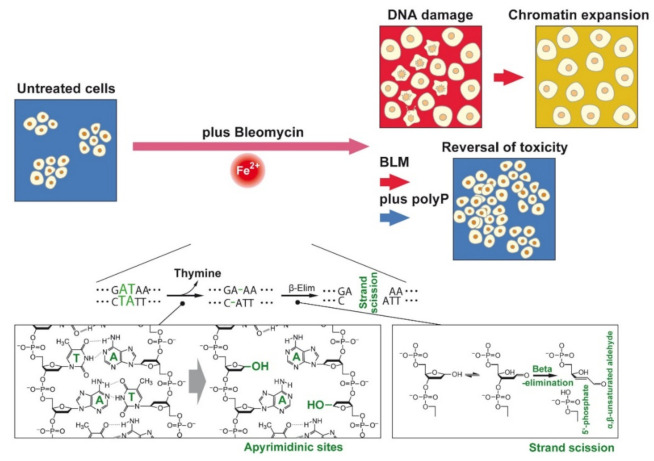
(Above) Survey about the target of the manuscript. BLM is added to untreated A549 cells. During the incubation with the drug the cells undergo DNA damage followed by chromatin expansion. This effect can be (at least partially) blocked by polyP. (Below) During the incubation with BLM the integrity of DNA is broken. After activation of BLM by O_2_ thymine bases are released from the DNA, especially at sites adjacent to a purine. At those sites, apyrimidinic sites, β-elimination occurs that results in the formation of α, β-unsaturated aldehyde and 5′-phosphate DNA strand termini (according to [21]).

One of the most interesting molecular biological effects of BLM is its effect on the DNA strand integrity. Our group described [11] that BLM attacks thymidine in DNA and other polydeoxyribonucleotides by releasing free thymine and leaving the aldehyde group in the DNA. This reaction can be accelerated by adding reducing as well as oxidizing agents. In addition, the bithiazole group with its cationic moiety could be traced as a functionally essential group that binds to DNA. Higher BLM concentrations were found to transfer the modified DNA into oligothyminic or athyminic nucleic acids and ultimately cause strand scissions [12] (Figure 2). Later this reaction was defined by the elucidation that BLM, after activation in the presence of O_2_ and its binding to BLM-Fe(II), is forming with O_2_ a complex. The formed BLM-Fe(II)-OO accepts an electron and H^+^, a process during which the complex is converted to BLM-Fe(III)-OOH [13,14,15]. In the presence of O_2_ a direct strand break occurs under formation of 3′-phosphoglycolate and 5′-phosphate ends and the release of the base, usually it is thymine. The process of Fe(II) to Fe(III) is reversible, prompting the authors to call BLM a quasi-enzyme [16]. This term was subsequently used by other authors as well [17]. The reactivation of Fe(III) to (Fe(II) like in BLM [18] is also seen in traditional enzymes, like in ferritin [19]. The specific action of BLM for thymine residues in DNA has been exploited for the sequencing of the lac operator [20].

BLM causes also DNA damage in intact human cells [22]. Interestingly, BLM acts at specific DNA sites within the cells, like in the repetitive centromeric alphoid DNA region, tandemly repeated sequences [22], or telomeric sequences [23]. In addition, BLM cleaves at transcription factors and linker regions between nucleosomes [24], as well as actively transcribed genes [25]. In turn BLM can be applied for the determination of chromatin structure and the formation of chromatid breaks [26].

Among the major adverse side effects of BLM in patients is a severe lung toxicity which induces remodeling of lung architecture and loss of pulmonary function, most likely due to the induction of fibrosis [27]. This progression is the result of endothelial and interstitial capillary edema, pneumocyte type II proliferation and surfactant overproduction, pneumocyte type II necrosis and surfactant release. As a consequence fibroblast proliferation and trans-differentiation occurs as a result of activated fibroblasts [28,29]. The effect of pneumocyte type II cells has been studied also in vitro, applying the A549 cells [30] or BEAS-2B cells [31]. While A549 cells are adenocarcinomic human alveolar basal epithelial cells, BEAS-2B cells are non-tumorigenic lung epithelial cells. These cells respond to 10 µM BLM with increased inflammation and apoptosis, as well as migration, an effect that can be abolished by curcumin. In contrast, MLE12/15, likewise alveolar type II pneumocytes [32], were described to show a decrease in the proliferation rate most likely via induction of apoptosis and cell cycle arrest after treatment with BLM [33]. Furthermore, these cells show an increased size of the nuclei, which has been interpreted as a process of cell senescence. Finally, BLM causes an increase in the number of airway secretory cells, paralleled with an upregulation of mucin production [34].

BLM is intracellularly inactivated by the enzyme BLM hydrolase, which reduces the biological activity of the drug. This enzyme hydrolyzes the carboxamide bond of the β-aminoalanine moiety on the BLM molecule to a carboxylic acid (Figure 1). The enzyme was isolated from rat l iver [35] and characterized as a cysteine proteinase. Later the respective gene was cloned and expressed [36]. An in vitro assay was described [37] which could be subsequently applied as a BLM-sensitivity test for staging of human carcinomas in the head and neck region [38]. The lowest activity of BLM hydrolase was measured in biopsies of highly differentiated as well as moderately differentiated squamous cell carcinomas, suggesting that the drug causes the strongest effect in those carcinomas.

Major symptoms of the presently storming COVID-19 pandemics are the severe acute respiratory distress syndrome reflected by an idiopathic pulmonary fibrosis [39]. Since the corresponding animal studies have been performed with BLM animal models of pulmonary fibrosis it appears not to be appropriate at all to apply BLM for an antifibrotic therapy. Recently, a physiological natural polymer, polyphosphate (polyP), synthesized in larger amounts in blood platelets and also present in a soluble form in the circulating blood, has been identified as a molecule that binds with selectivity to the receptor-binding domain (RBD) of the S (spike)-protein of SARS-CoV-2 [40,41]. In turn, binding of the RBD to the host cell receptor angiotensin-converting enzyme 2 (ACE2) [42] was sensitively blocked. Interestingly, data indicate that ACE2 attenuates BLM-induced lung fibrosis by reversing the reduction of local ACE2 and suppressing the elevation of idiopathic pulmonary fibrosis [43].

PolyP was disclosed as an anti-fibrotic polymer that reduces intestinal inflammation and fibrosis via downregulation of the expression of inflammation- and fibrosis-associated molecules, like interleukin 1β [44,45]. In the present study, and by using A549 cells, we show that the BLM-caused reduction of cell viability–most likely due to an increased DNA damaging effect and a subsequent chromatin expansion process–can be sensitively prevented by co-incubation with polyP (Figure 2). Molecular evidence is presented by qRT-PCR (real-time polymerase chain reaction) that this favorable property is due to the previously observed morphogenetic activity caused by polyP, which fires the expression of BLM hydrolase [46].

## 2. Materials and Methods

### 2.1. Materials

Na-polyphosphate (Na-polyP) with an average chain length of 40 P_i_ units (polyP_40_) was obtained from Chemische Fabrik Budenheim (Budenheim, Germany). BLM, containing a mixture of BLM-A_2_ (~60%) and BLM-B_2_ (~30%), was a gift from Mack (Illertissen, Germany).

### 2.2. A549 Cells

A549 cells (#86012804, Sigma, Taufkirchen, Germany; ATCC #CCL-185), a human lung (carcinoma) line, were cultivated in Dulbecco’s modified Eagle’s medium (DMEM; Sigma) containing 10% fetal calf serum (FCS; Biochrom GmbH, Berlin, Germany) as described [47,48]. The cells were seeded at a density of 40,000 cells cm^−2^ (1 mL) in 8-well plates in a humidified atmosphere of 5% CO_2_ in air (37 °C). Every 3 to 4 days the medium/serum was replaced. If the cells were incubated with additional compounds for a longer period, half of the medium/serum was replaced after 3 days with new medium/serum containing the original components with the concentration used for the inoculum. All assays with polyP added additionally contained 5 mM CaCl_2_ to prevent chelation by the polymer [49]. BLM was dissolved in phosphate-buffered saline (PBS) and added at concentrations mentioned with the results.

### 2.3. BEAS-2B Cells

BEAS-2B cells a human bronchial epithelial cell line (#95102433; Sigma–Merck; ATCC #CRL-9609) were cultured in keratinocyte serum-free growth medium (#131-500A; Sigma-Aldrich) as described [50]. The studies were performed with BEAS-2B cells between passage 10 and passage 30. They were seeded at 40,000 cells cm^−2^ and then grown in 5% CO_2_ in air (37 °C) as outlined for the A549 cells.

### 2.4. Staining of the Cells: Immunocytochemistry

A549 cells were cultivated on sterile cover glass and treated with BLM and/or polyP for up to 4 days. The cells were fixed with 4% paraformaldehyde (10 min), permeabilized with 0.2% Triton X-100 (10 min). Then the cells [nuclei] were stained with DRAQ5 (#65-0880-92; Thermo Fisher Scientific, Dreieich, Germany) [51,52]. Alternatively the living cells were stained with Calcein-AM (#C1359, Sigma-Aldrich) [53].

The images were taken with a fluorescence microscope (Olympus, Tokyo, Japan) using a wavelength of 646 nm/662 nm (DRAQ5) and 495 nm (Calcein-AM). Where indicated Nomarski optics was used to contrast the light microscopical images. The nuclear sizes were obtained by application of ImageJ (Informer Technologies, Rijswijk, Zuid, The Netherlands).

### 2.5. Cytotoxicity Assays: MTT

The cells either A549 adenocarcinomic human alveolar basal epithelial cells, BEAS-2B cells which are immortalized but non-tumorigenic human cell, or human osteogenic sarcoma cells (SaOS-2 cells; Sigma #89050205) [53] were used. They were seeded on a 96-well plate at a cell density of 3000 per well for 72 h were reacted with MTT (thiazolyl blue tetrazolium bromide; #M2128, Sigma-Aldrich). After the development of the insoluble formazan product into a colored solution, the plates were read at 570 nm [54,55].

For the statistical analyses the Student’s *t* test was applied. The average values came from ten independent experiments. Values of *p* < 0.05 were considered statistically significant (*). The calculations were performed with the GraphPad Prism 7.0 software (GraphPad Software, La Jolla, CA, USA).

### 2.6. DNA Stand Break Assay

The Fast Micromethod was applied to determine DNA damage in A549 cells as described [56]. This system bases on the property of the specific fluorochrome dye PicoGreen (#P0990, Sigma) to form a stable complex with double-stranded DNA [57]. Increased breakage in DNA is reflected by a decrease of the fluorimetric signal which is proportionate to an increasing single-strand DNA breakage. The cells are lysed in the microplates in the presence of SDS (sodium dodecyl sulfate) containing PicoGreen. The DNA denaturation kinetics is recorded after addition of an alkaline NaOH–EDTA solution for 20 min at an excitation of 485 nm and an emission of 520 nm in a Fluoroscan II reader (Labsystems, Finland). The results are expressed by the strand scission factor (SSF) which is, for practical reasons, in a reciprocal way. In turn, a SSF × (−1) value from 0.1 to 1.0 indicates an increase in the extent of damaged DNA. The vales were calibrated towards calf thymus double-stranded DNA (#D4522, Sigma-Aldrich).

A549 cells were seeded at a density of 40,000 cells cm^−2^ in 8-well plates. After an incubation period of 3 days the cells were collected and used for the break determinations. Incubations with BLM, polyP or combinations are specified with the respective experiments. Five parallel assays were performed. The significance was calculated by ANOVA (by Bonferroni adjustment) program [58].

### 2.7. Quantitative Real-Time Polymerase Chain Reaction: BLM Hydrolase

A detailed description of the qRT-PCR (reverse transcriptase-PCR) procedure applied has been given before [59]. The A549 cells were exposed to polyP, BLM or the two compounds in co-incubation, or remained untreated (controls); the respective concentrations are given under “Results”. Cells were incubated in 8-well plates (40,000 cells cm^−2^) and incubated for 1 or 3 days. Then RNA was extracted, reverse-transcribed into cDNA using reverse transcriptase (Promega Corp., Madison, WI, USA) and an oligo(dT)_16_ primer (Promega). The amplified PCR products were obtained after 28–32 cycles (30 s at 94 °C, annealing for 45 s at 50 °C, primer extension for 30 s at 72 °C). For the amplification of the BLM hydrolase [60,61,62], the forward primer 5′-ACCAGCCCATTGACTTCC-3′ and the reverse primer 5′-TGTCCACCACCACTTCGT-3′ were used. The values were normalized with the expression levels of the reference housekeeping gene *GAPDH* (glyceraldehyde 3-phosphate dehydrogenase; NM_002046.3) with the primer pair Fwd, 5′-ACTTTGTGAAGCTCATTTCC-3′ and Rev, 5′-TTGCTGGGGCTGGTGGTCCA-3′. The amplifications were performed in triplicate in an iCycler (Bio-Rad, Hercules, CA, USA), and the mean C_t_ values and the efficiencies were calculated applying the iCycler software (Bio-Rad) [63]; the estimated PCR efficiencie s were 95–103%.

### 2.8. Statistical Analysis

Student t test was applied to perform comparisons between two groups. Values of *p* < 0.05 were considered statistically significant (*). The calculations were performed with the GraphPad Prism 7.0 software.

## 3. Results

### 3.1. Effect of BLM on Cell Growth

**Figure 3:**
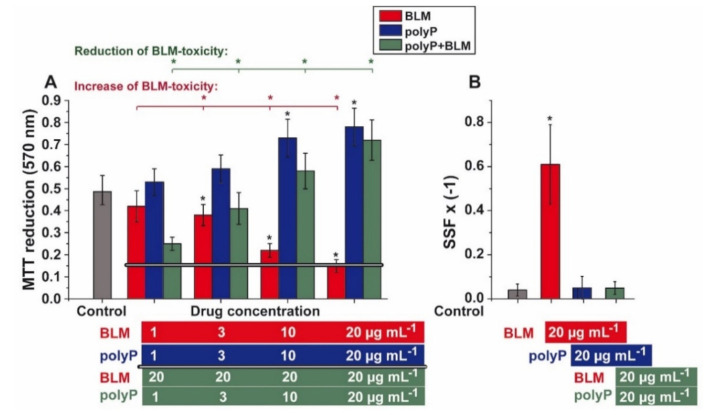
Effect of BLM and BLM together with polyP on (**A**) cell viability and (**B**) DNA integrity. (**A**) BLM was added at increasing concentrations to A549 cells resulting to an enhanced cell toxicity (red bars), incubation for for 72 h. The level reached at 20 µg mL^−1^ was used for the coincubation studies (horizontal line). In parallel, the effect of polyP was measured (blue bars). In co-incubation studies 20 µg mL^−1^ of BLM were co-incubated with increasing concentrations of polyP, starting with 1 µg mL^−1^; a significant reduction of cell toxicity was reached at polyP concentrations higher than 1 µg mL^−1^ (green bars); the incubation period was 72 h. The control level is given as grey bar. The values came from 10 parallel experiments; The significances within an individual group are indicated (* *p* < 0.01). (**B**) Level of DNA breaks in A549 cells. In the absence of any drug the level is low (grey bar). Addition of 20 µg mL^−1^ of BLM drastically increased the extent of DNA damage (red bar), while after addition of 20 µg mL^−1^ polyP (blue bar) or 20 µg mL^−1^ polyP together with 20 µg mL^−1^ of BLM (green bar) control levels were reached.

It is surprising that BLM does not cause complete inhibition of cell proliferation in vitro not only for tumor cells [64] but also for mesenchymal stem cells [65]. At a concentration of around 10 to 20 µg mL^−1^ a maximum is achieved with about 60% reduction of the proliferation rate. Based on this finding a BLM concentration of (usually) 20 µg mL^−1^ was applied for the A549 cell studies presented here. In the latter cell system (A549 cell) an ~70% reduction of the viability was obtained by using the MTT assay (Figure 3A). Lower concentrations of BLM are likewise inhibitory, starting significantly at a concentration of 3 µg mL^−1^ with ~20%. The same degree of inhibition was also found for human osteogenic sarcoma cells (SaOS-2 cells).

It was the aim of the present study to elucidate if the physiological, morphogenetically acting polymer polyP interferes with the growth of the cells. The soluble Na-polyP was used for the study, here a concentration of also 20 µg mL^−1^ was applied since this concentration has earlier been detected to cause a morphogenetic effect on cells in vitro, for example to induce mineralization in osteoblast-like SaOS-2 cells [66]. This concentration of polyP (20 µg mL^−1^) caused a 66% increase in A549 cell growth (Figure 3A). If increasing concentrations of this polymer are co-added to BLM (at 20 µg mL^−1^) a gradual decrease of BLM-caused cell toxicity is measured. At the level of 1 µg mL^−1^ of co-addition the reduction of BLM-caused toxicity (BLM, 20 µg mL^−1^) comes to ~60%, a value that further increases close to a value seen in the polyP assays alone.

### 3.2. Effect of BLM and polyP Alone and in Combination on BEAS-2B Cell Viability

**Figure 4:**
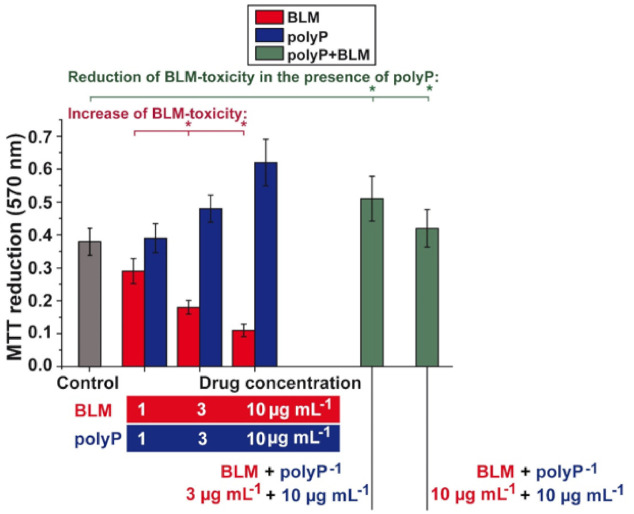
Effect of BLM and polyP on cell viability of BEAS-2B cells. While BLM is reducing the cell viability in the MTT assay (red bars), polyP is enhancing the viability (blue bars). If these two compounds are added together to the cultures, at the indicated concentrations, not only an elimination of the inhibitory activity of BLM is seen, but even an increase of viability is measured in the assays (green columns). (* p < 0.01).

Parallel to the viability studies with A549 cells, the effects of BLM and of polyP on BEAS-2B cells have been determined as well (Figure 4). A related inhibition/stimulation pattern is seen between the two cell types. For BEAS-2B cells a reduction of the cell viability is seen for BLM at 1 µg L^−1^ to 70.7%, at 3 µg L^−1^ to 43.9%, and at 10 µg L^−1^ to 26.8%, respectively. In contrast, polyP increase cell viability at concentrations >3 µg mL^−1^ to 126% (3 µg mL^−1^) or to 163% (10 µg mL^−1^). In combination, addition of BLM together with polyP to the BEAS-2B cells: If polyP is added at a concentration of 10 µg L^−1^ to the cultures, exposed to 3 µg L^−1^ BLM, the degree of viability increased to 134%. Likewise, cultures incubated with 10 µg L^−1^ BLM, together with 10 µg L^−1^ of polyP, showed still a higher viability with 111%; the controls (without BLM or polyP) were set to 100% (Figure 4).

### 3.3. DNA Strand Breaks Caused by BLM

The effect of BLM on DNA integrity was measured with the Fast Micromethod Assay [56,58]. The strand scission factor (SSF), a value for the level of DNA scissions, is given–for practical reasons–in a reciprocal way. In consequence a higher value reflects a more severe DNA damage.

The A549 cells were–after seeding–incubated for 3 days in the presence of those drug concentrations that have also been used for the co-incubation experiments in the MTT study. In the controls, only a low level of DNA breaks is detected with an SSF × (−1) value of 0.04 (Figure 3B). This level increased drastically if the DNA of the cells incubated with 20 µg mL^−1^ of BLM was analyzed. In those cells the value increases significantly to an SSF × (−1) value of 0.61. In contrast, cells incubated with 20 µg mL^−1^ of polyP alone, or in combination with 20 µg mL^−1^ of BLM reached again control values, of around 0.05.

### 3.4. Severe effects of BLM on Cell Morphology

**Figure 5:**
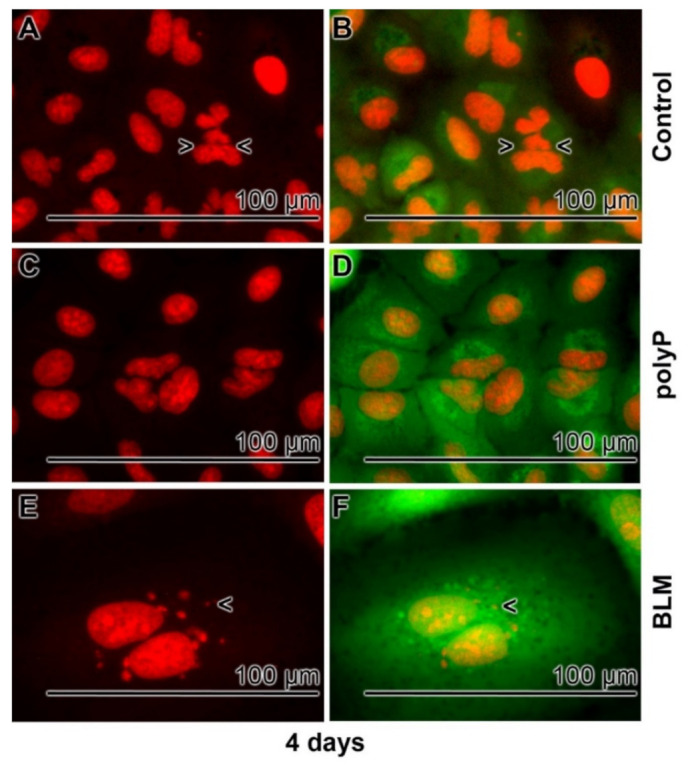
Enlargement of A549 cells after exposure to BLM; 4 days incubation period. The cells are stained with DRAQ5 for a visualization of the nuclei (left panel), or with Calcein-AM (viable cells; right panel). (**A**,**B**) In normal cultures the nuclei have a size of ~12 µm, similar to (**C**,**D**) those measured in cells treated with polyP (20 µg mL^−1^). Often nuclei division figures are seen (>,<). (**E**,**F**) In contrast, the volume of the cells in BLM treated cultures increases markedly; seldom fragmentation signs are seen (<).

Untreated A549 cells grow for 3 or 4 days in the medium/serum as described under “Materials and Methods”. During this period the cells attach to the culture dish and form a unilayer organization. The cell size is ~25–30 µm in diameter harboring a nucleus of ~12 µm (Figure 5A,B); 4 days incubation. The sizes of the nuclei were assessed by ImageJ. If the cultures were exposed to 20 µg mL^−1^ of polyP the morphology does not change and also the number of nuclear division figures remain the same between the controls and the polyP treated samples (Figure 5C,D). This aspect is very different in samples treated with 20 µg mL^−1^ of BLM (Figure 5E,F). In those treated assays the nuclear size increases to ~20–25 µm in diameter; a similar enlargement of the nuclei has been reported recently [33]. The nuclei do not show marked signs of apoptotic fragmentation, blebbing, shrinkage, fragmentation or chromatin condensation [67] suggesting that this phenotype reflects decreased G_0_/G_1_ transition, perhaps paralleled with an increased S and G_2_/M phase dynamics. Rarely also fragmentation signs are seen around the cell nuclei [68]. The increase in cell/nulcei volume is very pronounced and the expansion measured from 898 ± 154 µm^3^ in the controls to 5324 ± 894 µm^3^ of the drug treated A549 cells; a 6-fold increase.

### 3.5. Prevention of Nuclear Enlargement in BLM Treated Cells During Co-Incubation with *polyP*

**Figure 6:**
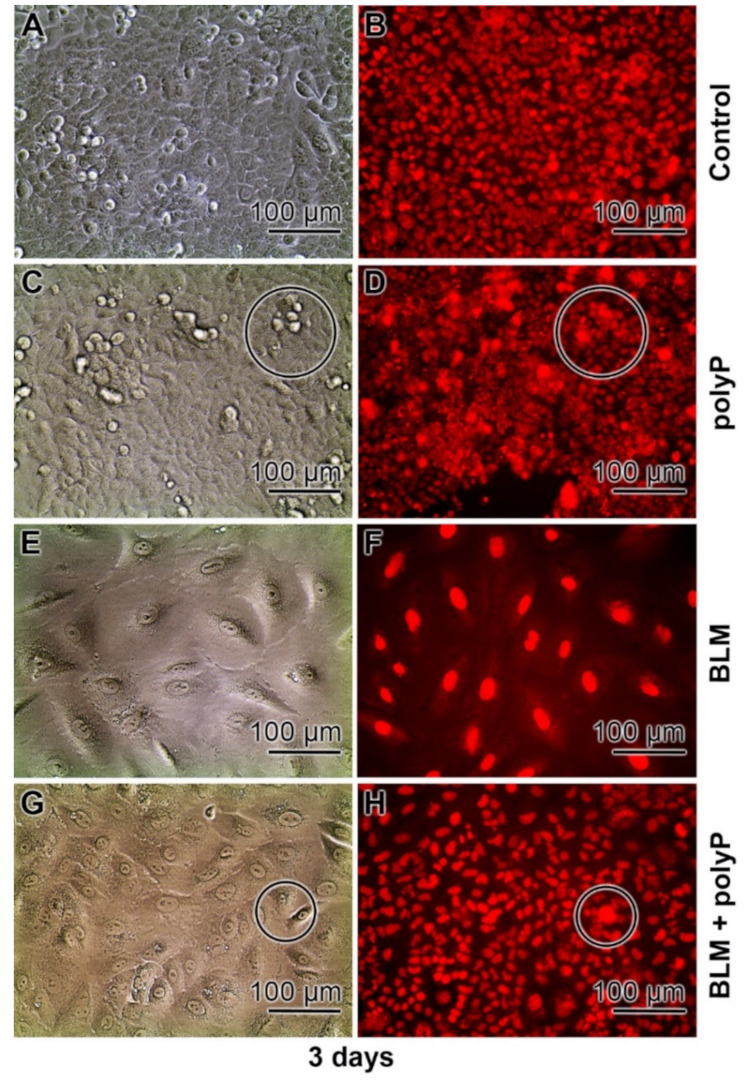
Prevention of chromatin/nuclear expansion of A549 cells by polyP; 3 days incubation, and inspection by Nomarski optics (left panel) and immunofluorescence microscopy (DRAQ5). (**A**,**B**) In the controls the cells are arranged in a cobblestone pattern. (**C**,**D**) After treatment of the cells with polyP (20 µg mL^−1^) the cells show frequently a three dimensional organization patter (circles). (**E**,**F**) BLM treated culture (20 µg mL^−1^) with the large nuclei. (**G**,**H**) Co-incubation of the cells with BLM (20 µg mL^−1^) and polyP (20 µg mL^−1^). Occasionally, patches with a three-dimensional growth of the cells exist (circles).

The nuclear expansion can be prevented by co-incubation with polyP (Figure 6). In this histological series the aspects of the cells, the adherent cells were inspected by light microscopy (Nomarski optics), were visualized after staining with DRAQ5 to highlight the nuclei by immunofluorescence. In this series the cells were incubated for 3 days. Again, in the controls the A549 cells are tightly attached to each other under formation of a cobblestone pattern of adjoining cells, as described [69]; Figure 6A. DRAQ5 staining indicated that the cells are growing perfectly in a single cell pattern (Figure 6B). The density of the cell layer in the polyP (20 µg mL^−1^) treated assays is similarly dense (Figure 6C); also reflected after staining with DRAQ5. However, often a three-dimensional growth of the cells is visible (Figure 6D). Such a pattern is normal for A549 cells but is intensified if the cells are triggered to synthesize collagen [70]. Again, in a strong contrast the BLM treated assays (20 µg mL^−1^) show a much larger cell nucleus, with even ~25 µm in diameter after the 3 days incubation period (Figure 6E,F). The light microscopical images show a tight interaction of the cells with each other; the morphology is more spherical. In the central co-incubation series the A549 cells were exposed to BLM (20 µg mL^−1^) together with polyP (20 µg mL^−1^). Under those conditions, the morphology of the cells and the nuclei in them show normal sizes (Figure 6G,H).

### 3.6. Effect of Sequential Application of BLM and *polyP*

**Figure 7:**
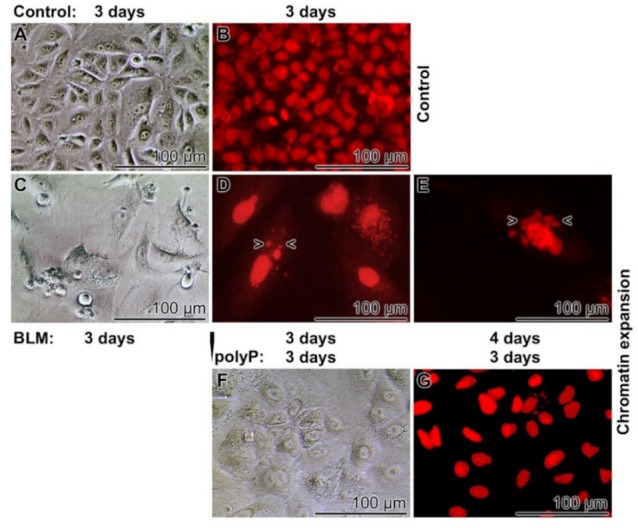
Sequential application of BLM (20 µg mL^−1^), followed by polyP (20 µg mL^−1^), to A549 cells; (**A**,**C**,**F**), Nomarski optics; the other cells are stained with DRAQ5. (**A**,**B**) In the controls the cells show a cobblestone single-layer growth pattern. (**C** to **E**) Exposure of the cells to BLM causes an enlargement of the cell nuclei during the 3 days or 4 day incubation period. Some fragmentations are seen (>,<). (**F**,**G**) A transfer of the cells from BLM to an exclusively polyP medium/serum and continuation of incubation for 3 days results only in a slight reduction of the nuclear size.

In a comparative study it was tested if the BLM (20 µg mL^−1^)-caused increase in cell volume is reversed by a secondary application of polyP (20 µg mL^−1^) to the cells (Figure 7). Again, in the controls without the two compounds the cells grow to a smooth layer after 3 days of incubation. The cells are arranged in a cobblestone pattern (Figure 7A,B). In the presence of BLM the pattern of cell arrangement becomes more irregular (Figure 6C) and the nuclei reaches sizes of ~25 to 28 µm in diameter (Figure 7D). After an extension of the incubation to 4 days some nuclear fragmentation is occasionally visible (Figure 7E). If the BLM-treated cultures (for 3 days) are transferred to a BLM-free medium/serum with polyP (20 µg mL^−1^) for a period of 3 days the size of the nuclei decreases slightly to 23.3 ± 8.4 µm (Figure 7F,G). Those cells remain in a viable state for at least three additional incubation days (data not shown).

### 3.7. Time Dependent Deleterious Influence of BLM on of the polyP Rescue Activity: A549 Cells and BEAS-2B Cells

**Figure 8:**
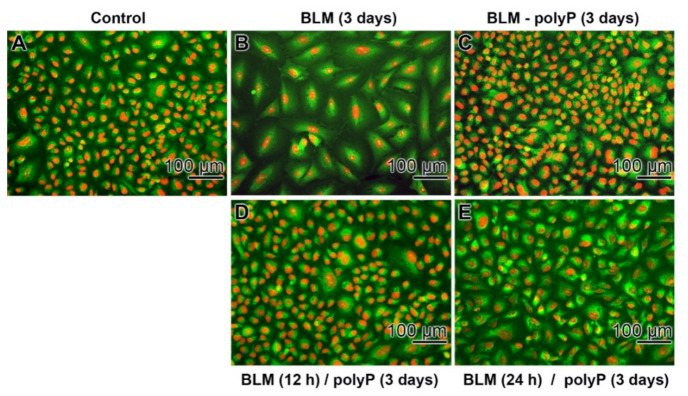
Different duration of incubation/preincubation of A549 cells with BLM (20 µg mL^−1^) to determine the rescue activity of polyP (20 µg mL^−1^); the cells are stained with DRAQ5 (staining of nuclei) together with Calcein-AM (viable cells). In the controls (**A**) absence of any compound, (**B**) effect of BLM alone, and (**C**) on BLM together with polyP during the 3 days incubation period the different sizes of the cells are obvious. (**D**) Exposure of the cells with BLM for 12 h followed, after washing, by an incubation with polyP for 3 days. (**E**) The preincubation period with BLM was extended to 24 h, followed by an incubation with polyP (3 days).

As outlined above, the rescue effect of polyP on the BLM-caused expansion of the cells become irreversible if the drug exposure had been 3 days (Figure 7). Therefore, in a further series of experiments the duration of the BLM exposure was shortened to 12 h and 24 h, respectively (Figure 8). In the controls (without drugs) and in the assays with BLM together with polyP the cells are small (Figure 8A,C). In contrast, in assays with BLM alone the size increases strongly (Figure 8B). However, if the incubation schedule is changed and BLM is added to the cells for 12 h or for 24 h, followed by a 3 days incubation with polyP, the sizes of the cells turn by to smaller ones (Figure 8D,E).

**Figure 9:**
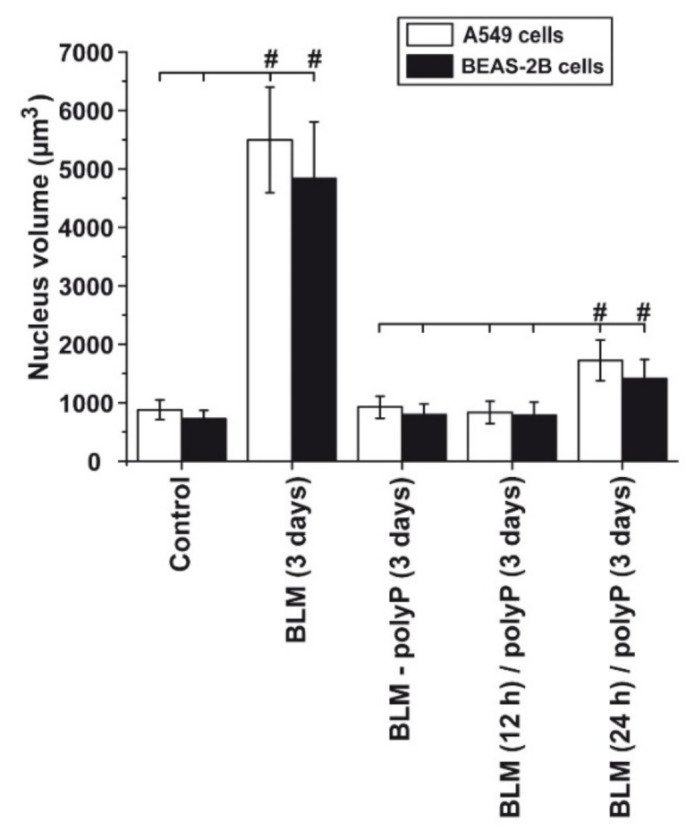
Quantitative assessment of the effect of BLM on both A549 cells and BEAS-2B cells during a different pre-incubation period. In the controls, either in the absence of any drug (control) or in the presenc of BLM alone, a strong increase of the BLM-treated cell nuclei occur. However, if the cells are pre-incubated for 12 h or for 24 h with BLM, followed by a post-incubation with polyP the increase of the nuclei is only marginal. 100 nuclei were measured per assay; #, *p* < 0.01; against the corresponding values in the groups (marked with a horizontal line).

A quantitative assessment of the rescue effect of polyP was performed both with A549 cells and with BEAS-2B cells (Figure 9). The size of the nuclei in BEAS-2B cells is slightly smaller than in A549 cells. The graph shows that addition of BLM to both cells caused the drastic enlarging effect from about 800 µm^3^ (controls) to ~5000 µm^3^ (BLM-treated cultures for 3 days) during the 3 days incubation period. However, if in a pre-incubation period the drug BLM exposure was shortened to 12 h or 24 h followed by the addition of polyP (in the absence of BLM) the size of the nuclei increased only slightly to to ~800 µm^3^ (12 h BLM) or ~1500 µm^3^ (24 h BLM). These data show that the aberrant effect of BLM is much reduced during a shorter incubation period.

### 3.8. Effect of BLM and *polyP* Alone or in Combination on BLM Hydrolase Gene Expression

**Figure 10:**
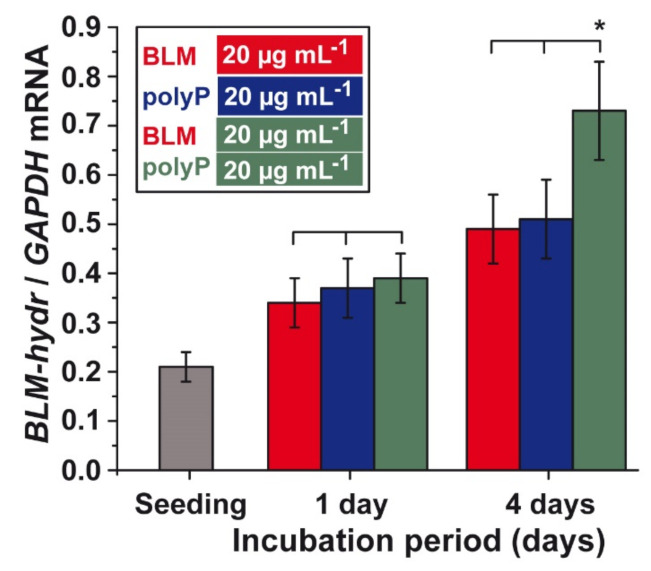
Gene steady-state-expression of the *BLM hydrolase* (BLM-hydr.) after incubation of A549 cells with BLM (20 µg mL^−1^) or polyP (20 µg mL^−1^) alone, or in combination. The cells were incubated for 1 day or 4 days with the components. Then RNA was extracted, reverse transcribed and amplified for *BLM hydrolase* expression or of the one for the house keeping gene *GAPDH*. Standard errors of the means (SEM) are indicated (n = 5 experiments per time point). Within one incubation time point the significance has been calculated; * *p* < 0.05.

A549 cells were incubated with BLM (20 µg mL^−1^) or polyP (20 µg mL^−1^) alone or in combination and the steady-state expression of the gene encoding the BLM hydrolase was measured by qRT-PCR. The exposure of the cells to the single components does not cause a significant difference in the expression level within one group. After 1 day of incubation, an approximately 70% increase of the expression levels is seen (Figure 10). However, if the incubation period is extended to 4 days the values increased again by 40% for the cells exposed to a single component. If the two compounds (BLM and polyP) are added simultaneously to the cultures a significant increase in the BLM hydrolase expression level of 3.5-fold is measured (Figure 10).

## 4. Discussion

**Figure 11:**
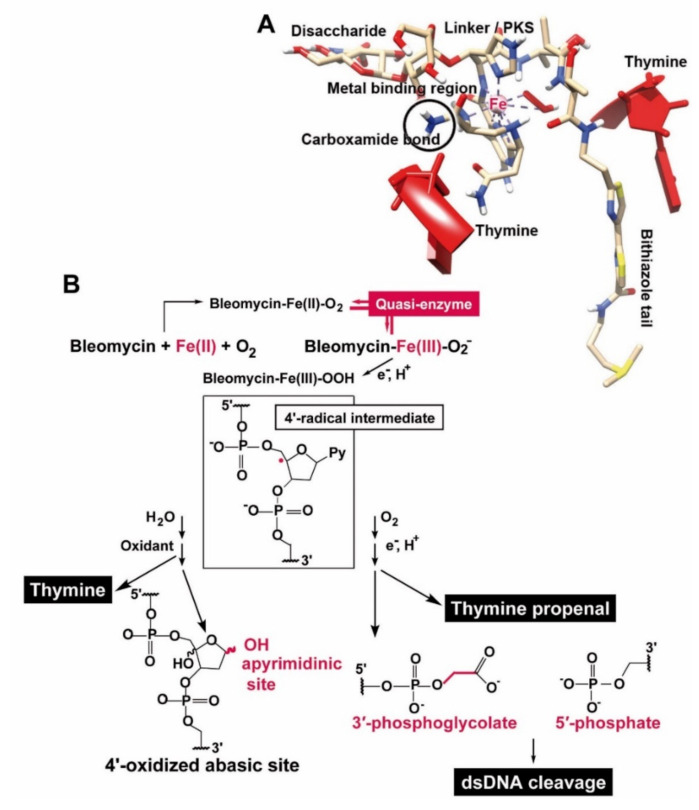
BLM-caused DNA strand scission. (**A**) Applying the NMR structure of HO_2_-Co(III)bleomycin A_2_ bound to d(GAGCTC) as a template (PDB file; [21]) the positions of the staggered thymine bases in double-stranded DNA are indicated. Besides of showing the segments within BLM (see Figure 1) also the binding positions of BLM to DNA can be postulated. Since the BLM hydrolase is opening the carboxamide bond of the β-aminoalanine moiety [73] this site is crucial for the binding of BLM. (**B**) The sequential process of DNA strand scission involves a 4′-radical intermediate, followed by the formation of a apyrimidinic site and the subsequent formation of the 3′-phosphoglycolate and the 5′-phosphate termini at the DNA strand breaks (modified according to [15,16]).

BLM is a member of a first class of DNA splitting drugs that remove preferentially thymine from double- and single-stranded DNA [11]. After the discovery that this drug is intracellularly activated by iron ions [13] a clear view on the mode of action of BLM became possible. DNA is cleaved either through the Fe(II) drug complex together with O_2_ in two subsequent steps, or via a Fe(III) drug complex together with H_2_O_2_ in one step. The oxidation state of Fe in BLM is reversible [15] (Figure 11). Based on this property BLM was termed a quasi-enzyme [16], suggesting a relationship to a nuclease cleaving at an apyrimidinic site within the DNA [71,72]. The BLM hydrolase is cleaving the carboxamide bond of the β-aminoalanine moiety [73] resulting in a loss of biological activity of the drug. This amide group is proposed to bind to DNA [thymine] [16]. It has been proposed that binding of BLM to DNA occurs via the hydrogen atom of the amide group in the carboxyl amide moiety, which forms a hydrogen bond with the 2-keto group of the thymine. This keto group of thymine is exposed to the narrow groove of the DNA double helix. For both single- and double-stranded DNA, and depending on the availability of O_2_, the DNA is attacked via a 4′-radical intermediate a process which causes the releases of thymine from the DNA und the formation of an apyrimidinic site. From there DNA damage starts and allows the 3′-phosphoglycolate and the 5′-phosphate termini to form [15,74].

Applying BLM for the application in the A549 cell system the effect of BLM on the quantitative insertion of DNA strand breakage was studied. In the first set of experiments the effect of BLM on cell viability was tested. Based on earlier studies a concentration of BLM of 20 µg mL^−1^ was chosen; at this dose an incomplete inhibition is usually achieved and a plateau is obtained. Likewise, a concentration of 20 µg mL^−1^ of polyP was used since this concentration was found to be close to the maximum dose required for a detection the morphogenetic effect of polyP [66]. Using the highly sensitive assay for the detection of DNA strand breakages in intact cell systems, the Fast MicroMethod [56], it was disclosed that 20 µg mL^−1^ of BLM reduces cell growth by ~70%. In parallel to this effect the number of DNA strand scissions caused by this drug increased sharply. Even though it could be anticipated that co-incubation with polyP might reduce the toxic effect of BLM [75], due to the metabolic energy fueling property of polyP, in form of ATP, it came as a surprise that at these concentrations an almost total reduction of the toxicity both with respect to cell growth and DNA damage was reached.

To clarify if the cells are impaired by BLM through necrosis or apoptosis, the cells were inspected after staining with DRAQ5 (to visualize the nuclei) or with Calcein-AM (whole cell morphology) or by light microscopy using Nomarski optics. Again the finding that the size of the cells in BLM-treated cultures dramatically increased from a volume seen in the controls, with ~900 µm^3^, to ~5300 µm^3^ in drug treated A549 cells as well as in BEAS-2B cells was unexpected. A similar effect in A549 cells has also been reported previously [47]. Already due to this increase in the size of the nucleus a process of apoptosis can be excluded as the prevalent underlying cause [67]. It remains to be elucidated if in other cell lines an apoptotic cell death in response to BLM is predominant [76,77]. However, occasionally signs of apoptotic fragmentation, like blebbing, are detected. Additionally the process of ferroptosis might also occur, which reflects a type of programmed cell death dependent on iron and which is also characterized by the accumulation of lipid peroxides [78]. A further process, especially in cells exposed to antineoplastic drugs, including those causing DNA strand scission [79], or during suppressing of HIV [80] is (partially) caused by members of the Schlafen family of proteins [81]. However, in spite of the fact that the cell motility is also affected, no data have been published that suggest an increase of the cell nucleus during these cell reactions.

DNA damage in cells is often paralleled with a gradual chromatin compaction phase after rapid expansion [82]. This dynamic packaging of chromatin, from a compact to a diffuse state, was taken as an evidence for homologous recombination or for unrepaired DNA double-strand breaks [83]. During the process of chromatin expansion the heterochromatin fraction facilitates the extrusion of damaged DNA to the periphery of the heterochromatin where the DNA repair is completed. It has been proposed that dynamic nuclear expansion is regulated by distinct factors [84] and is driven by the nuclear envelope besides of the chromatin state [85]. In addition, induced genomic instability contributes to an increased nuclear size [86].

The adverse effect of BLM on cell growth and DNA integrity can be effectively prevented by co-incubation with polyP. In contrast, a sequential administration of BLM to the cells first, followed by polyP, has no influence on the change in the nuclear size. A preventing effect of free radicals, produced by polyP, can–at the present state of knowledge–be excluded. No radical mediating effect caused by the polymer itself has been reported yet.

An efficient detoxification mechanism of BLM in cancer cells is the hydrolytic cleavage of the carboxamide bond at the β-aminoalanine moiety on the BLM molecule to a carboxylic acid via the BLM hydrolase [35]. This enzyme, a neutral cysteine protease, reduces not only the DNA damaging activity of BLM in vitro but also in vivo [12]. The gene [36] has been identified in human lymphomas as well as in patients with atopic dermatitis and psoriasis [62] at different levels. In turn, the obvious assumption was that after addition of polyP to the BLM-treated cells an induction of the gene encoding for the BLM hydrolase will take place. The inducing, morphogenetic features and properties of polyP, e.g., the induction of collagen or alkaline phosphatase, has been well documented [46]. The steady-state-expression data of *BLM hydrolase* confirmed this expectation. However, the fact that the gene is inducibly expressed by polyP only during an exposure to BLM was not anticipated. This finding informs us that the assessability of the transcription factors involved in the induction of this gene is more complex. The promoter of the BLM hydrolase has a characteristic structure that lacks consensus transcriptional sequences such as TATA box or CCAAT box, as can also be seen in other housekeeping genes [87], but might be prone to enhancers/enhancer genetic control [88].

With regard to a potential application of polyP in the treatment with BLM it should be emphasized that this polymer with a physiological chain length of around 40 P_i_ units, as used in this study, does not show any adverse effects by influencing blood coagulation [89,90], in contrast to bacterial long-chain polyP [91].

## 5. Conclusions

The major outcome of the study is the documentation that the known toxicity of BLM to human lung (carcinoma) A549 cells can be abolished by co-incubation with polyP. This finding will contribute to a further rational application of this drug during the initiation of lung toxicity, fibrosis, also with respect to a use of BLM in the treatment schedule in COVID-19 patients. In one in vivo study, using the dextran sodium sulfate (DSS)-induced chronic colitis model in mice, it had been disclosed that polyP ameliorate fibrosis in vivo in the mouse model [92]. The gathered data showed that poly P suppresses intestinal inflammation and fibrosis through downregulating of the expression of inflammation- and fibrosis-associated molecules in the intestinal epithelium [44,92]. Furthermore, it had been published that the CXCL14/CXCR4 chemokine axis is involved in the progression and/or activation of fibroblasts during initiation of pulmonary fibrosis in the lung [93]. In tunr, one therapeutic starting treatment could be the application of 2-aminopurine [94].

With respect to a potential therapeutic intervention controlling COVID-19 disorders a modulation with the transforming growth factor β had been discussed [39]. This combination schedule appears not to be a theoretical but is boosted by recent findings that demonstrated polyP being an effective blocking agent of binding of SARS-CoV-2 proteins to the cellular receptor ACE2 [40,41]. Even more, this physiological polymer induces mucin expression in the respiratory airways system at concentrations that show the anti-corona viral activity [95]. A proposed application in the form of a spray [41] that can be combined with other drugs [96] has been established.

## Data Availability

The data presented in this study are available in this article.
